# Reviewer acknowledgement 2014

**DOI:** 10.1186/1479-7364-8-2

**Published:** 2014-01-24

**Authors:** Vasilis Vasiliou

**Affiliations:** 1 Department of Pharmaceutical Sciences, Molecular Toxicology and Environmental Health Sciences Program, University of Colorado Anschutz Medical Center, Aurora, CO 80045, USA

## Abstract

**Contributing reviewers:**

The Editors of *Human Genomics* would like to thank all our reviewers who have contributed to the journal in volume 7 (2013).

## 

Osborne Almeida

Germany

Christina Aquilante

United States of America

Donald Backos

United States of America

Sanjay Bharti

United States of America

Joseph Borg

Malta

Elspeth Bruford

United Kingdom

Elizabeth Bryda

United States of America

John Carpenter

United States of America

Giorgio Casari

Italy

Sebahattin Cirak

United States of America

Robin Dowell

United States of America

Lynnette Robin Ferguson

New Zealand

Alonso Fernandez-Guasti

Mexico

Ana Teresa Freitas

Portugal

Baruch Frenkel

United States of America

Dexiang Gao

United States of America

Paraskevi Goggolidou

United Kingdom

Jakob Grove

Denmark

Pei-Ing Hwang

Taiwan

Peilin Jia

United States of America

Kalpana Kannan

United States of America

Kiyoshi Kawakami

Japan

Sok Kean Khoo

United States of America

Rick Kittles

United States of America

Bernd Klaus

Germany

Michael Knudsen

Denmark

Chee Seng Ku

Singapore

Poh-San Lai

Singapore

Jun Li

United States of America

Peng Li

United States of America

Jerry Lingrel

United States of America

Donna Maglott

United States of America

Jose Luis Millan

United States of America

Jonathan Mudge

United Kingdom

Maria Nastase Mannila

Sweden

Daniel Nebert

United States of America

George Patrinos

Greece

Sirkku Peltonen

Finland

Tzu Lip Phang

United States of America

David Pollock

United States of America

Stan Pounds

United States of America

Richard Radcliffe

United States of America

Michael Lee Robinson

United States of America

Jadranka Sertic

Croatia

Imran Shah

United States of America

Mark Shriver

United States of America

Alessio Squassina

Italy

Shivalingappa Swamynathan

United States of America

Ronald Trent

Australia

Panagiotis Tsonis

United States of America

Evita van den Herik

Netherlands

Bruce Weir

United States of America

Zhi-Hong Wen

Taiwan

Carsten Wiuf

Denmark

Yuliang Wu

Canada

Dmitri Zaykin

United States of America

Guojie Zhang

China

Verena Zuber

Norway

